# 5-Ethyl-3-(3-fluoro­phenyl­sulfon­yl)-2-methyl-1-benzofuran

**DOI:** 10.1107/S1600536811015443

**Published:** 2011-04-29

**Authors:** Hong Dae Choi, Pil Ja Seo, Byeng Wha Son, Uk Lee

**Affiliations:** a Department of Chemistry, Dongeui University, San 24 Kaya-dong Busanjin-gu, Busan 614-714, Republic of Korea; b Department of Chemistry, Pukyong National University, 599-1 Daeyeon 3-dong, Nam-gu, Busan 608-737, Republic of Korea

## Abstract

In the title compound, C_17_H_15_FO_3_S, the fluoro­phenyl ring makes a dihedral angle of 76.11 (5)° with the mean plane of the benzofuran fragment. In the crystal, mol­ecules are linked by weak inter­molecular C—H⋯O hydrogen bonds and C—H⋯π inter­actions.

## Related literature

For the biological activity of benzofuran compounds, see: Aslam *et al.* (2009 ▶); Galal *et al.* (2009 ▶); Khan *et al.* (2005 ▶). For natural products with benzofuran rings, see: Akgul & Anil (2003 ▶); Soekamto *et al.* (2003 ▶). For structural studies of related 5-alkyl-3-(4-fluoro­phenyl­sulfon­yl)-2-methyl-1-benzofurans, see: Choi *et al.* (2010**a* ▶,b* ▶,*c* ▶).
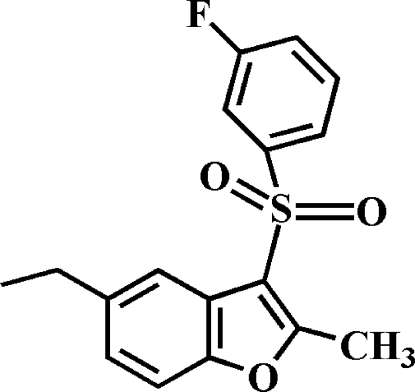


## Experimental

### 

#### Crystal data

C_17_H_15_FO_3_S
*M* *_r_* = 318.35
Orthorhombic, 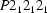

*a* = 8.4395 (1) Å
*b* = 11.3701 (2) Å
*c* = 15.3559 (2) Å
*V* = 1473.52 (4) Å^3^
*Z* = 4
Mo *K*α radiation
μ = 0.24 mm^−1^
*T* = 173 K
0.35 × 0.25 × 0.16 mm

#### Data collection

Bruker SMART APEXII CCD diffractometer
Absorption correction: multi-scan (*SADABS*; Bruker, 2009 ▶) *T*_min_ = 0.920, *T*_max_ = 0.962
14844 measured reflections
3665 independent reflections
3453 reflections with *I* > 2σ(*I*)
*R*_int_ = 0.035

#### Refinement

*R*[*F* ^2^ > 2σ(*F* ^2^)] = 0.032
*wR*(*F* ^2^) = 0.077
*S* = 1.09
3665 reflections
200 parameters
H-atom parameters constrained
Δρ_max_ = 0.23 e Å^−3^
Δρ_min_ = −0.30 e Å^−3^
Absolute structure: Flack (1983 ▶), 1555 Friedel pairs
Flack parameter: −0.01 (6)

### 

Data collection: *APEX2* (Bruker, 2009 ▶); cell refinement: *SAINT* (Bruker, 2009 ▶); data reduction: *SAINT*; program(s) used to solve structure: *SHELXS97* (Sheldrick, 2008 ▶); program(s) used to refine structure: *SHELXL97* (Sheldrick, 2008 ▶); molecular graphics: *ORTEP-3* (Farrugia, 1997 ▶) and *DIAMOND* (Brandenburg, 1998 ▶); software used to prepare material for publication: *SHELXL97*.

## Supplementary Material

Crystal structure: contains datablocks global, I. DOI: 10.1107/S1600536811015443/bh2350sup1.cif

Structure factors: contains datablocks I. DOI: 10.1107/S1600536811015443/bh2350Isup2.hkl

Supplementary material file. DOI: 10.1107/S1600536811015443/bh2350Isup3.cml

Additional supplementary materials:  crystallographic information; 3D view; checkCIF report

## Figures and Tables

**Table 1:** Hydrogen-bond geometry (Å, °) *Cg* is the centroid of the C2–C7 benzene ring.

*D*—H⋯*A*	*D*—H	H⋯*A*	*D*⋯*A*	*D*—H⋯*A*
C6—H6⋯O2^i^	0.95	2.49	3.206 (2)	133
C13—H13⋯O3^ii^	0.95	2.51	3.395 (2)	155
C9—H9*A*⋯*Cg*^iii^	0.99	2.68	3.625 (2)	159

Symmetry codes: (i) 
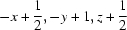
; (ii) 
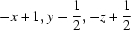
; (iii) 
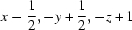
.

**Fig. 1.**
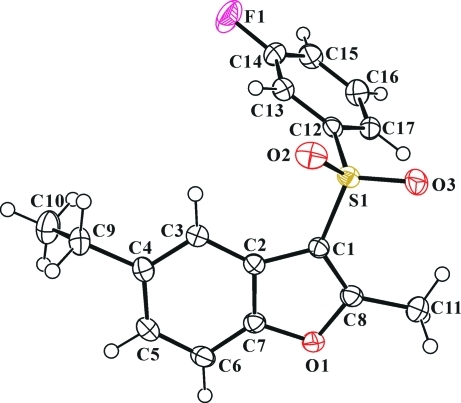
The molecular structure of the title compound with displacement ellipsoids drawn at the 50% probability level. H atoms are presented as a small spheres of arbitrary radius.

**Fig. 2.**
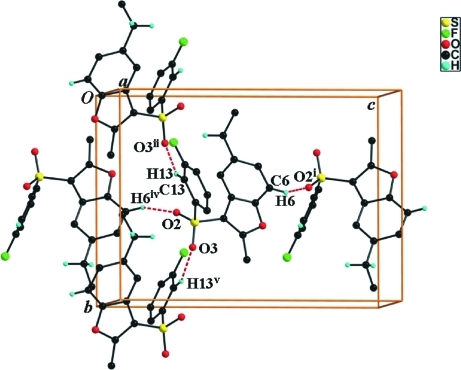
A view of the C–H···O interactions (dotted lines) in the crystal structure of the title compound. [Symmetry codes: (i) -x+1/2, -y+1, z+1/2; (ii) -x+1, y-1/2, -z+1/2; (iv) -x+1/2, -y+1, z-1/2; (v) -x+1, y+1/2, -z+1/2].

**Fig. 3.**
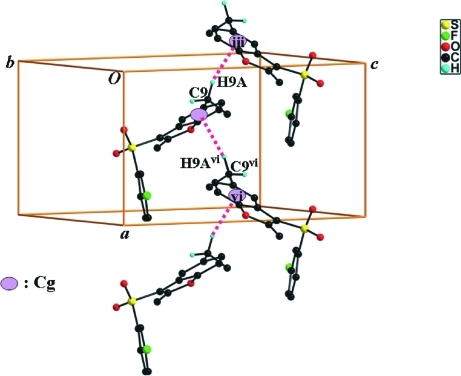
A view of the C–H···π interactions (dotted lines) in the crystal structure of the title compound. [Symmetry codes: (iii) x-1/2, -y+1/2, -z+1; (vi) x+1/2, -y+1/2, -z+1].

## supplementary crystallographic information

### Comment

Many compounds having a benzofuran skeleton exhibit interesting biological properties such as antibacterial and antifungal, antitumor and antiviral, and antimicrobial activities (Aslam *et al.*, 2009; Galal *et al.*, 2009; Khan *et al.*, 2005). These compounds occur in a wide range of natural products (Akgul & Anil, 2003; Soekamto *et al.*, 2003). As a part of our ongoing study of the substituent effect on the solid state structures of 5-alkyl-3-(4-fluorophenylsulfonyl)-2-methyl-1-benzofuran analogues (Choi *et al.*, 2010**a*,*b*,c*), we report herein on the molecular and crystal structures of the title compound.

The title compound crystallizes in the non-centrosymmetric space group *P*2_1_2_1_2_1_ in spite of having no asymmetric C atoms.

In the title compound (Fig. 1), the benzofuran unit is essentially planar, with a mean deviation of 0.017 (1) Å from the least-squares plane defined by the nine constituent atoms. The 3-fluorophenyl ring makes a dihedral angle of 76.11 (5)° with the mean plane of the benzofuran fragment. The crystal packing (Fig. 2) is stabilized by weak intermolecular C–H···O hydrogen bonds; the first one between a benzene H atom and the O atom of the sulfonyl group (Table 1; C6–H6···O2^i^), and the second one between a 3-fluorophenyl H atom and the O atom of the sulfonyl (Table 1; C13–H13···O3^ii^). The crystal packing (Fig. 3) is further stabilized by intermolecular C–H···π interactions between a methylene H atom of the ethyl group and the benzene ring (Table 1; C9–H9A···Cg^iii^, Cg is the centroid of the C2···C7 benzene ring).

### Experimental

77% 3-Chloroperoxybenzoic acid (560 mg, 2.5 mmol) was added in small portions to a stirred solution of 5-ethyl-3-(3-fluorophenylsulfanyl)-2-methyl-1-benzofuran (320 mg, 1.2 mmol) in dichloromethane (40 mL) at 273 K. After being stirred at room temperature for 6 h, the mixture was washed with saturated sodium bicarbonate solution and the organic layer was separated, dried over magnesium sulfate, filtered and concentrated at reduced pressure. The residue was purified by column chromatography (hexane-ethyl acetate, 4:1 *v*/*v*) to afford the title compound as a colorless solid [yield 71%, m.p. 395-396 K; R_f_ = 0.51 (hexane-ethyl acetate, 2:1 *v*/*v*)]. Single crystals suitable for X-ray diffraction were prepared by slow evaporation of a solution of the title compound in diisopropyl ether at room temperature.

### Refinement

All H atoms were positioned geometrically and refined using a riding model, with C–H = 0.95 Å for aryl, 0.99 Å for methylene and 0.98 Å for methyl H atoms, respectively. *U*_iso_(H) = 1.2*U*_eq_(C) for aryl and methylene H atoms, and 1.5*U*_eq_(C) for methyl H atoms.

### Crystal data

**Table:**

C_17_H_15_FO_3_S	*D*_x_ = 1.435 Mg m^−^^3^
*M**_r_* = 318.35	Melting point: 395 K
Orthorhombic, *P*2_1_2_1_2_1_	Mo *K*α radiation, λ = 0.71073 Å
Hall symbol: P 2ac 2ab	Cell parameters from 6176 reflections
*a* = 8.4395 (1) Å	θ = 2.2–27.6°
*b* = 11.3701 (2) Å	µ = 0.24 mm^−^^1^
*c* = 15.3559 (2) Å	*T* = 173 K
*V* = 1473.52 (4) Å^3^	Block, colourless
*Z* = 4	0.35 × 0.25 × 0.16 mm
*F*(000) = 664	

### Data collection

**Table:**

Bruker SMART APEXII CCD diffractometer	3665 independent reflections
Radiation source: rotating anode	3453 reflections with *I* > 2σ(*I*)
graphite multilayer	*R*_int_ = 0.035
Detector resolution: 10.0 pixels mm^-1^	θ_max_ = 28.3°, θ_min_ = 2.2°
φ and ω scans	*h* = −11→11
Absorption correction: multi-scan (*SADABS*; Bruker, 2009)	*k* = −14→15
*T*_min_ = 0.920, *T*_max_ = 0.962	*l* = −19→20
14844 measured reflections	

### Refinement

**Table:**

Refinement on *F*^2^	Secondary atom site location: difference Fourier map
Least-squares matrix: full	Hydrogen site location: difference Fourier map
*R*[*F*^2^ > 2σ(*F*^2^)] = 0.032	H-atom parameters constrained
*wR*(*F*^2^) = 0.077	*w* = 1/[σ^2^(*F*_o_^2^) + (0.0404*P*)^2^ + 0.1728*P*] where *P* = (*F*_o_^2^ + 2*F*_c_^2^)/3
*S* = 1.09	(Δ/σ)_max_ = 0.001
3665 reflections	Δρ_max_ = 0.23 e Å^−^^3^
200 parameters	Δρ_min_ = −0.30 e Å^−^^3^
0 restraints	Absolute structure: Flack (1983), 1555 Friedel pairs
0 constraints	Flack parameter: −0.01 (6)
Primary atom site location: structure-invariant direct methods	

### Fractional atomic coordinates and isotropic or equivalent isotropic displacement parameters (Å^2^)

**Table:**

	*x*	*y*	*z*	*U*_iso_*/*U*_eq_	
S1	0.55279 (4)	0.61633 (3)	0.30198 (2)	0.02203 (9)	
F1	0.83613 (14)	0.24805 (9)	0.20382 (9)	0.0530 (3)	
O1	0.45021 (12)	0.62846 (9)	0.55130 (7)	0.0262 (2)	
O2	0.43231 (13)	0.56682 (11)	0.24756 (7)	0.0326 (3)	
O3	0.59979 (14)	0.73710 (10)	0.29031 (8)	0.0320 (3)	
C1	0.49455 (16)	0.59635 (12)	0.40936 (9)	0.0208 (3)	
C2	0.41587 (16)	0.49312 (12)	0.44346 (9)	0.0203 (3)	
C3	0.36195 (16)	0.38604 (13)	0.40976 (9)	0.0221 (3)	
H3	0.3794	0.3663	0.3504	0.027*	
C4	0.28224 (17)	0.30910 (13)	0.46495 (10)	0.0241 (3)	
C5	0.25768 (18)	0.34013 (14)	0.55252 (11)	0.0278 (3)	
H5	0.2020	0.2870	0.5891	0.033*	
C6	0.31114 (19)	0.44473 (15)	0.58750 (11)	0.0278 (3)	
H6	0.2944	0.4648	0.6468	0.033*	
C7	0.39063 (17)	0.51841 (13)	0.53065 (10)	0.0227 (3)	
C8	0.51210 (18)	0.67416 (13)	0.47591 (10)	0.0246 (3)	
C9	0.2197 (2)	0.19327 (14)	0.43146 (11)	0.0300 (4)	
H9A	0.2258	0.1931	0.3671	0.036*	
H9B	0.1066	0.1860	0.4479	0.036*	
C10	0.3089 (2)	0.08760 (15)	0.46610 (15)	0.0425 (5)	
H10A	0.4204	0.0929	0.4488	0.064*	
H10B	0.2625	0.0155	0.4422	0.064*	
H10C	0.3016	0.0860	0.5298	0.064*	
C11	0.5782 (2)	0.79444 (14)	0.48421 (12)	0.0351 (4)	
H11A	0.6645	0.7941	0.5269	0.042*	
H11B	0.4949	0.8484	0.5036	0.042*	
H11C	0.6191	0.8203	0.4276	0.042*	
C12	0.72448 (17)	0.52843 (13)	0.29036 (9)	0.0210 (3)	
C13	0.70954 (19)	0.41921 (14)	0.25199 (11)	0.0272 (3)	
H13	0.6099	0.3904	0.2327	0.033*	
C14	0.8463 (2)	0.35391 (15)	0.24302 (12)	0.0322 (4)	
C15	0.9925 (2)	0.39204 (16)	0.27131 (11)	0.0327 (4)	
H15	1.0838	0.3439	0.2649	0.039*	
C16	1.00295 (19)	0.50192 (15)	0.30918 (11)	0.0312 (3)	
H16	1.1028	0.5301	0.3288	0.037*	
C17	0.86942 (18)	0.57153 (13)	0.31887 (10)	0.0251 (3)	
H17	0.8769	0.6473	0.3445	0.030*	

### Atomic displacement parameters (Å^2^)

**Table:**

	*U*^11^	*U*^22^	*U*^33^	*U*^12^	*U*^13^	*U*^23^
S1	0.02206 (16)	0.02353 (17)	0.02051 (16)	0.00299 (14)	0.00042 (13)	0.00420 (14)
F1	0.0469 (6)	0.0286 (6)	0.0835 (9)	0.0008 (5)	0.0124 (6)	−0.0213 (6)
O1	0.0279 (5)	0.0269 (5)	0.0239 (5)	−0.0037 (5)	0.0046 (4)	−0.0048 (4)
O2	0.0275 (6)	0.0449 (7)	0.0254 (6)	0.0034 (5)	−0.0062 (5)	0.0003 (5)
O3	0.0394 (6)	0.0228 (5)	0.0337 (6)	0.0054 (5)	0.0075 (5)	0.0086 (5)
C1	0.0188 (6)	0.0215 (7)	0.0221 (7)	−0.0002 (5)	0.0015 (5)	0.0002 (5)
C2	0.0174 (6)	0.0217 (7)	0.0218 (7)	0.0023 (5)	0.0003 (5)	0.0016 (5)
C3	0.0209 (6)	0.0237 (7)	0.0216 (7)	0.0011 (6)	−0.0012 (5)	0.0008 (6)
C4	0.0195 (7)	0.0246 (8)	0.0282 (8)	−0.0002 (5)	−0.0018 (6)	0.0039 (6)
C5	0.0241 (8)	0.0310 (8)	0.0282 (8)	−0.0023 (6)	0.0045 (6)	0.0048 (6)
C6	0.0264 (7)	0.0334 (9)	0.0236 (8)	−0.0009 (6)	0.0056 (6)	0.0002 (6)
C7	0.0195 (6)	0.0247 (7)	0.0239 (7)	0.0007 (6)	0.0008 (5)	−0.0026 (6)
C8	0.0223 (7)	0.0266 (8)	0.0250 (7)	−0.0001 (6)	0.0026 (6)	−0.0011 (6)
C9	0.0302 (8)	0.0258 (8)	0.0341 (9)	−0.0071 (6)	−0.0021 (7)	0.0023 (7)
C10	0.0443 (10)	0.0253 (9)	0.0579 (13)	−0.0019 (7)	−0.0031 (9)	0.0033 (8)
C11	0.0406 (9)	0.0279 (8)	0.0368 (9)	−0.0094 (7)	0.0056 (8)	−0.0075 (7)
C12	0.0226 (6)	0.0204 (7)	0.0198 (7)	0.0005 (5)	0.0026 (5)	0.0043 (5)
C13	0.0262 (7)	0.0272 (8)	0.0281 (8)	−0.0038 (6)	0.0031 (6)	−0.0007 (6)
C14	0.0366 (9)	0.0231 (8)	0.0369 (9)	0.0007 (6)	0.0087 (8)	−0.0039 (6)
C15	0.0277 (7)	0.0301 (8)	0.0402 (9)	0.0067 (7)	0.0070 (7)	0.0047 (7)
C16	0.0228 (7)	0.0337 (8)	0.0372 (9)	−0.0014 (6)	−0.0036 (7)	0.0019 (7)
C17	0.0266 (7)	0.0229 (7)	0.0259 (8)	−0.0012 (6)	−0.0003 (6)	0.0008 (6)

### Geometric parameters (Å, °)

**Table:**

S1—O2	1.4315 (12)	C9—C10	1.515 (2)
S1—O3	1.4405 (12)	C9—H9A	0.9900
S1—C1	1.7356 (14)	C9—H9B	0.9900
S1—C12	1.7692 (14)	C10—H10A	0.9800
F1—C14	1.3484 (19)	C10—H10B	0.9800
O1—C8	1.3723 (18)	C10—H10C	0.9800
O1—C7	1.3853 (18)	C11—H11A	0.9800
C1—C8	1.360 (2)	C11—H11B	0.9800
C1—C2	1.4466 (19)	C11—H11C	0.9800
C2—C7	1.386 (2)	C12—C13	1.380 (2)
C2—C3	1.399 (2)	C12—C17	1.389 (2)
C3—C4	1.391 (2)	C13—C14	1.379 (2)
C3—H3	0.9500	C13—H13	0.9500
C4—C5	1.406 (2)	C14—C15	1.378 (2)
C4—C9	1.509 (2)	C15—C16	1.381 (3)
C5—C6	1.381 (2)	C15—H15	0.9500
C5—H5	0.9500	C16—C17	1.385 (2)
C6—C7	1.383 (2)	C16—H16	0.9500
C6—H6	0.9500	C17—H17	0.9500
C8—C11	1.483 (2)		
			
O2—S1—O3	119.86 (7)	C4—C9—H9B	108.9
O2—S1—C1	107.58 (7)	C10—C9—H9B	108.9
O3—S1—C1	108.72 (7)	H9A—C9—H9B	107.7
O2—S1—C12	107.50 (7)	C9—C10—H10A	109.5
O3—S1—C12	107.48 (7)	C9—C10—H10B	109.5
C1—S1—C12	104.70 (7)	H10A—C10—H10B	109.5
C8—O1—C7	106.68 (12)	C9—C10—H10C	109.5
C8—C1—C2	107.81 (13)	H10A—C10—H10C	109.5
C8—C1—S1	126.72 (12)	H10B—C10—H10C	109.5
C2—C1—S1	125.46 (11)	C8—C11—H11A	109.5
C7—C2—C3	119.21 (13)	C8—C11—H11B	109.5
C7—C2—C1	104.59 (13)	H11A—C11—H11B	109.5
C3—C2—C1	136.17 (14)	C8—C11—H11C	109.5
C4—C3—C2	118.62 (14)	H11A—C11—H11C	109.5
C4—C3—H3	120.7	H11B—C11—H11C	109.5
C2—C3—H3	120.7	C13—C12—C17	122.18 (14)
C3—C4—C5	119.75 (14)	C13—C12—S1	118.45 (12)
C3—C4—C9	120.68 (14)	C17—C12—S1	119.36 (11)
C5—C4—C9	119.56 (14)	C14—C13—C12	116.78 (15)
C6—C5—C4	122.70 (15)	C14—C13—H13	121.6
C6—C5—H5	118.7	C12—C13—H13	121.6
C4—C5—H5	118.7	F1—C14—C15	118.59 (15)
C5—C6—C7	115.76 (15)	F1—C14—C13	118.16 (16)
C5—C6—H6	122.1	C15—C14—C13	123.25 (16)
C7—C6—H6	122.1	C14—C15—C16	118.35 (15)
C6—C7—O1	125.35 (14)	C14—C15—H15	120.8
C6—C7—C2	123.95 (14)	C16—C15—H15	120.8
O1—C7—C2	110.65 (13)	C15—C16—C17	120.67 (15)
C1—C8—O1	110.26 (13)	C15—C16—H16	119.7
C1—C8—C11	134.90 (15)	C17—C16—H16	119.7
O1—C8—C11	114.83 (13)	C16—C17—C12	118.77 (14)
C4—C9—C10	113.49 (14)	C16—C17—H17	120.6
C4—C9—H9A	108.9	C12—C17—H17	120.6
C10—C9—H9A	108.9		
			
O2—S1—C1—C8	−140.71 (14)	C2—C1—C8—O1	0.08 (17)
O3—S1—C1—C8	−9.51 (16)	S1—C1—C8—O1	179.27 (10)
C12—S1—C1—C8	105.13 (15)	C2—C1—C8—C11	−178.45 (17)
O2—S1—C1—C2	38.35 (14)	S1—C1—C8—C11	0.7 (3)
O3—S1—C1—C2	169.55 (12)	C7—O1—C8—C1	−0.53 (16)
C12—S1—C1—C2	−75.81 (13)	C7—O1—C8—C11	178.32 (13)
C8—C1—C2—C7	0.40 (16)	C3—C4—C9—C10	109.92 (17)
S1—C1—C2—C7	−178.81 (11)	C5—C4—C9—C10	−71.0 (2)
C8—C1—C2—C3	178.42 (16)	O2—S1—C12—C13	−14.67 (14)
S1—C1—C2—C3	−0.8 (2)	O3—S1—C12—C13	−144.95 (12)
C7—C2—C3—C4	1.0 (2)	C1—S1—C12—C13	99.55 (13)
C1—C2—C3—C4	−176.79 (15)	O2—S1—C12—C17	164.54 (12)
C2—C3—C4—C5	0.0 (2)	O3—S1—C12—C17	34.25 (14)
C2—C3—C4—C9	179.12 (13)	C1—S1—C12—C17	−81.25 (13)
C3—C4—C5—C6	−0.7 (2)	C17—C12—C13—C14	0.0 (2)
C9—C4—C5—C6	−179.80 (15)	S1—C12—C13—C14	179.16 (13)
C4—C5—C6—C7	0.3 (2)	C12—C13—C14—F1	−178.39 (15)
C5—C6—C7—O1	178.16 (14)	C12—C13—C14—C15	0.9 (3)
C5—C6—C7—C2	0.8 (2)	F1—C14—C15—C16	178.19 (15)
C8—O1—C7—C6	−176.83 (14)	C13—C14—C15—C16	−1.1 (3)
C8—O1—C7—C2	0.81 (16)	C14—C15—C16—C17	0.4 (3)
C3—C2—C7—C6	−1.5 (2)	C15—C16—C17—C12	0.5 (2)
C1—C2—C7—C6	176.93 (14)	C13—C12—C17—C16	−0.7 (2)
C3—C2—C7—O1	−179.17 (11)	S1—C12—C17—C16	−179.84 (12)
C1—C2—C7—O1	−0.74 (16)		

### Hydrogen-bond geometry (Å, °)

**Table:**

Cg is the centroid of the C2–C7 benzene ring.

**Table:**

*D*—H···*A*	*D*—H	H···*A*	*D*···*A*	*D*—H···*A*
C6—H6···O2^i^	0.95	2.49	3.206 (2)	133
C13—H13···O3^ii^	0.95	2.51	3.395 (2)	155
C9—H9A···Cg^iii^	0.99	2.68	3.625 (2)	159

Symmetry codes: (i) −*x*+1/2, −*y*+1, *z*+1/2; (ii) −*x*+1, *y*−1/2, −*z*+1/2; (iii) *x*−1/2, −*y*+1/2, −*z*+1.
